# Effects of different drying methods on physical properties and anthocyanin and volatile compound contents of black sweet corn (*Zea mays* L. *Saccharata* Sturt)

**DOI:** 10.3389/fnut.2025.1682022

**Published:** 2025-09-26

**Authors:** Pengyue Wang, Xue Chen, Xiqi Wei, Bingquan Xiong, Xiaomei Pan, Juhong Bai, Zidan Li, Xiangbo Xu

**Affiliations:** ^1^Chengdu Agricultural College, Chengdu, China; ^2^Sichuan Tourism University, Chengdu, China

**Keywords:** drying, color, rehydration, anthocyanin, volatile compounds, black sweet corn

## Abstract

**Background:**

Black sweet corn is a new corn variety that offers nutritional advantages; however, high water content makes it difficult to preserve. Drying is a widely employed postharvest processing technique to enhance shelf-life and economic value of agricultural products through moisture reduction.

**Methods:**

In this study, the effects of hot-air drying (HD), vacuum drying (VD), freeze drying (FD), and microwave drying (MD) on corn rehydration, color, texture, and anthocyanin and volatile compound contents were investigated.

**Results:**

It was observed that the VD products maintained the texture followed by the FD and MD products. The HD products exhibited poor ready-to-eat performance. All the drying methods exhibited significant impacts on the color of dehydrated black sweet corn, the L* and b* values of the fresh corn samples were significantly improved by the FD and MD treatment. The LC–MS/MS analysis of anthocyanin components showed that the FD and MD treatments could effectively mitigate anthocyanin losses during dehydration. A total of 67 volatile aroma components were identified using HS-SPME-GC–MS, and the number of substances (OAV > 1) having a significant effect on the flavor of the corn products were 24 (VD), 27 (FD), 21 (MD), 27 (HD), and 31 (fresh). The orthogonal partial least squares-discriminant analysis revealed linalool, (e)-2-nonenal, eucalyptol, 1-octen-3-ol, and estragole as the discriminatory differential (VIP > 1) aroma components of corn before and after drying. Furthermore, the relative contents of these five components were higher in the FD samples than in the other samples, followed by the MD samples.

**Conclusion:**

This study revealed the effects of different drying methods on the black sweet corn quality and laid the foundation for the selection of applicable drying methods to obtain ideal drying quality of black sweet corn.

## 1 Introduction

Maize (*Zea mays* L. *Saccharata* Sturt) is one of the top three global food crops and a major food source for humans and domestic animals (1). Considerable research efforts dedicated to novel cultivar development and quality enhancement have propelled the rapid expansion of the fresh sweet corn industry (2, 3). However, insufficient research on postharvest storage and processing technologies remains a key barrier to value chain extension for fresh sweet corn. Fresh sweet corn is vulnerable to microbial damage and difficult to preserve at ambient temperatures due to high water and sugar contents and weak epidermis (4). Sweet corn exhibits high postharvest perishability, with shelf life limited to 2–3 days at ambient temperatures and 3–5 days at 5 °C storage. The narrow harvest window (typically 5 days field-holding period) presents significant storage and marketing challenges for growers (5, 6).

Currently, the sweet corn market predominantly caters to fresh consumption, with a limited range of processed products available, including fresh produce (5), canned goods (2), crispy snacks (1), and beverages (6). This limitation is particularly pronounced in the case of new corn varieties, where the availability of processed products is even more restricted. Consequently, the existing selection fails to meet consumer demand for diverse and convenient options. Thus, research focused on the storage and processing of black sweet corn is of considerable practical and economic importance. This research focuses on “Heitianyu 13,” an innovative anthocyanin-rich sweet corn cultivar, which received approval from China’s national variety registration system in 2021. It has been extensively cultivated across several provinces in China, including Sichuan, Guangdong, and Tianjin, among others. Despite its commercial importance, there is a notable lack of published research on its postharvest storage and processing characteristics.

Anthocyanins are highly beneficial to human health owing to their various properties, such as antioxidative and anti-inflammatory properties. In addition, anthocyanins have been classified as a potent functional food (7). However, anthocyanins can be easily degraded with a change in various conditions, such as pH, temperature, and light. Therefore, reducing anthocyanin degradation is crucial for purple corn preservation (8).

Existing studies have measured the anthocyanin levels of dried purple corn (9), purple waxy corn (8), purple potato (10), black glutinous rice bran (7), sea buckthorn berries (11), and sea buckthorn pomace (12) using spectrophotometry. However, spectrophotometry exhibits limited sensitivity and cannot differentiate individual anthocyanin monomers. Zhou et al. (13) employed high-performance liquid chromatography (HPLC) to investigate anthocyanin variations in mulberry fruits before and after drying, a method that provides better separation. Nevertheless, anthocyanins remain the defining advantageous component of black sweet corn compared to conventional and other sweet corn cultivars. Thus, more effective detection methods are warranted. The liquid chromatography–tandem mass spectrometry (LC–MS/MS) is considered an effective bio-analytical method for the qualitative and quantitative analysis of anthocyanins in food (14, 15). Therefore, we quantitatively analyzed the anthocyanins in black sweet corn using LC–MS/MS, aiming to thoroughly elucidate the effects of drying methods on anthocyanins of black sweet corn.

Drying or dehydration—one of the oldest food preservation methods—removes moisture from the food and inhibits proliferation of food spoilage-causing bacteria, yeasts, and molds (16). When properly executed, this process effectively preserves food with good color, aroma, texture, and nutritional properties, particularly bioactive compounds (17). In addition, dehydrating the food reduces food weight and volume, thereby minimizing storage and transportation costs and enhancing processing potential (8). Several drying techniques have been developed for processing and preserving food samples (1, 7, 8, 12, 18). Hot-air drying (HD) utilizes high-speed airflow and convective heat transfer on the sample’s surface to facilitate moisture migration from the interior to the exterior. This technique offers advantages, such as cost-effectiveness, controllability, and ease of use. However, it may cause the loss of active constituents in the dried finished product (12). Vacuum drying (VD) can largely maintain the original flavor and stability of active ingredients in food. It has the advantages of ease of use, low drying temperature, low oxygen content in the drying chamber, and wide applicability. However, VD has limitations, such as low thermal energy utilization rate and low drying efficiency (18). Freeze drying (FD) is considered a suitable method for drying thermally sensitive pigments (7). FD is an emerging technology in the drying field in recent years. It utilizes sublimation to dehydrate the food and creates a low-temperature vacuum environment to preserve the color, shape, and nutritional ingredients of the food to a maximum extent. However, the high cost of FD equipment and the significant amount of drying time required to complete the process largely limit the industrial application potential of FD (12). Microwave drying (MD) utilizes the heat generated from the interaction of electromagnetic fields with food to facilitate drying. Heat is evenly distributed throughout the food, and heating is faster compared to conventional methods. A reduction in both energy consumption and operational costs is associated with the use of MD (19). This method provides rapid dehydration, but it can alter the color and flavor of the product (1, 8). However, the research on physicochemical properties and odor profile of black sweet corn using HD, MD, VD, and FD methods has not been reported. Therefore, this study aimed to investigate the effects of drying methods, namely, HD, MD, VD, and FD, on the physicochemical and nutritional properties of black sweet corn. The results could provide a basis for selecting applicable drying methods to obtain desirable quality of dried black sweet corn.

## 2 Materials and methods

### 2.1 Materials

Black sweet corns (Heitianyu 13) were provided by Chongzhou Science and Technology Park, Chengdu Agricultural College. The corns were harvested at the commercial maturity stage of 20 d after pollination. After harvesting, fresh black sweet corns were screened immediately and transported to the laboratory the same day. The corn kernels were removed from cobs by hand threshing. Then, the kernels were cleaned and drained. Afterward, a part of the kernels was frozen overnight at −20 °C for FD. Rest of the kernels were used for HD, MD, and VD. The original moisture content in the fresh black sweet corn was 75.34% ± 0.04% wet basis (w. b.).

### 2.2 Drying methods

For HD and VD, the dryer was run for 30 min at the beginning of the drying process to obtain a steady state, while for MD, this period was reduced to 5 min. For FD, the freeze dryer was pre-cooled for overnight before putting in the material. The material (500 g) was placed in the stainless steel tray or mesh bag of the dryer. The drying was discontinued when the moisture content fell below 10% (w. b.). All drying experiments were conducted in triplicate. The material was sealed and packed in aluminum foil pouch after cooling, and stored in the refrigerator for subsequent analysis.

HD was performed as described by Yao et al. (20). The HD equipment (DHG-9143BS-III) was obtained from Shanghai Xinmiao Medical Equipment Manufacturing Co. Ltd. (Shanghai, China). The drying medium temperature was set at 50 °C, the air speed was kept at 1 m/s, and the airflow direction was parallel to the drying material tray. The final moisture content of samples was 8.48% ± 0.23% W_w.b._, with a drying time of 18 h. MD was performed as described by Bhat et al. (21) with slight modification. The MD equipment (FEHCE502) was obtained from Shanghai Hecmac Hotel Equipment Manufacturing Co. Ltd. (Shanghai, China). The microwave power density was set at 3.6 W/g. The microwave was heated for 3 min and then cooled for 2 min. The material was weighed and dried to a constant weight. The final moisture content of samples was 8.10% ± 0.75% W_w.b._, with a total drying time of 0.75 h. FD was performed as described by Castillo-Gironés et al. (1) with slight modification. The FD equipment was obtained from Zhejiang Bohai Machinery Co. Ltd. (Zhejiang, China). The cold trap temperature and vacuum pressure in the drying chamber were set at −50 °C and 50 Pa, respectively. The final moisture content of samples was 8.86% ± 0.24% W_w.b._, with a drying time of 27 h. VD was performed as described by Pan and Cao (18). The black sweet corn samples (500 g) were spread in a single layer on sample trays, followed by drying in a benchtop vacuum dryer at 75 °C under −90 kPa vacuum. The VD equipment (DZF-6090) was obtained from Shanghai Yiheng Technology Instrument Co. Ltd. (Shanghai, China). The final moisture content of samples was 8.22% ± 0.20% W_w.b._, with a drying time of 24 h.

### 2.3 Color parameters

The color of grounded black sweet corn kernels was measured in terms of L*, a*, and b* by employing chromatic meter (CM-5, Konica Minolta, Inc., Japan). L* represents luminosity, a* and b* represent redness/greenness and yellowness/blueness, respectively. Color difference (Δ*E*) was calculated to estimate the color change in dried samples from that in their respective fresh samples using Equation 1.


(1) $$\mathrm{\Delta E}=\sqrt{{\left(L_{\mathrm{fs}}^{*}-L_{\mathrm{ds}}^{*}\right)}^{2}+{\left(a_{\mathrm{fs}}^{*}-a_{\mathrm{ds}}^{*}\right)}^{2}+{\left(b_{\mathrm{fs}}^{*}-b_{\mathrm{ds}}^{*}\right)}^{2}}$$

where the subscripts, fs, and ds, mean fresh and dehydrated samples, respectively.

### 2.4 Texture profile analysis (TPA)

The texture profile was estimated from the center of fresh and dehydrated black sweet corn kernels placed horizontally in a texture analyzer (Rapid TA+, Shanghai Tengba Instrument Technology Co. Ltd., Shanghai, China) based on the previously reported method (4). A P/2 cylindrical probe was used. The test parameters were configured as follows: pre-test speed of 1.00 mm s^−1^, test speed of 1.00 mm s^−1^, post-test speed of 1.00 mm s^−1^, strain of 30.0%, and trigger force of 10.0 g. Ten replicate tests were conducted for each experiment, and the mean value was calculated from the obtained data.

### 2.5 Rehydration ratio (RR)

The dehydrated samples were weighed, labeled, and fully immersed in a thermostatic water bath maintained at 30 °C. The kernels were retrieved at 10 min intervals (10, 20, 30, 40, 50, and 60 min) after immersion. Thereafter, the samples were carefully blotted using a qualitative filter paper to eliminate surface moisture prior to gravimetric measurement. This procedure was repeated in triplicate for each treatment group, and the RR was calculated using Equation 2.


(2) $$\mathrm{RR}=\frac{m_{b}}{m_{a}}$$

where *m_b_* and *m*_a_ denoted the weight of corn samples after absorption (g) and initial mass (g), respectively.

### 2.6 Qualitative and quantitative analysis of anthocyanins

The qualitative and quantitative analysis of anthocyanins was conducted using liquid chromatography–tandem mass spectrometry (LC–MS/MS) described by Lin et al. (15).

### 2.7 Volatile compounds analysis

Volatile compounds in corn were detected using a modified method reported by Zhang et al. (22). Briefly, 2.0 g of freshly ground sample was weighed in a 15 mL headspace bottle and equilibrated at 60 °C for 20 min. Then, volatiles were adsorbed on a 50/30 μm A DVB/CAR/PDMS extraction fiber (Supelco®, Bellefonte, PA, USA) for 40 min and desorbed for 5 min. A DB-WAX column (60 m × 0.25 mm × 0.25 μm, Agilent Technologies, Palo Alto, CA, USA) was used to separate volatiles, which were then analyzed by a gas chromatography system (GC, Agilent Technologies 7890B, Palo Alto, CA, USA) equipped with a triple quadrupole-mass spectrometer (TQ-MS, 8040 GC/MS Triple Quad, Shimadzu Corporation, Kyoto, Japan). Each sample was analyzed in triplicate.

The high-purity helium carrier gas flow rate was 1.0 mL min^−1^ with splitless injection mode. The temperature program was set as follows: initial temperature was set at 50 °C, followed by a ramping period from 50 to 85 °C at a rate of 10 °C min^−1^ and maintained for 1.5 min, which was then raised to 100 °C at a rate of 5 °C min^−1^ and held for 1 min, and sequentially raised to 175 °C at a rate of 2.5 °C min^−1^ and held for 1.5 min, followed by a final increase to 250 °C at a rate of 10 °C min ^−1^ and held for 3 min. The mass spectrometry conditions were as follow: the EI mode electronic energy was set at 70 eV with a mass scan range of 35.00–400.00 *m/z* and 230 and 280 °C were set as the ion source and quadrupole temperatures, respectively. The volatile compounds were identified by comparing the sample mass spectra with those in the NIST11 standard spectral library, and only compounds with a similarity index (SI) > 80 (maximum value 100) were considered for qualitative analysis. Semi-quantitative analysis was conducted using 4-methyl-2-pentanol as the internal standard to calculate the mass concentrations of the identified volatile components.

### 2.8 Statistical analysis

SIMCA software (Version 14.1, Umetrics Sweden) was utilized for conducting orthogonal partial least squares-discriminant analysis (OPLS-DA) to examine the variation in volatile aroma compounds among black sweet corn samples. OPLS-DA is a model that evaluates the explanatory power of *R*^2^(x), *R*^2^(Y), and *Q*^2^ fit parameters. The model was assessed using a permutation test with 200 iterations, obtaining *R*^2^ and *Q*^2^ values through permutation testing to ascertain if the model is prone to overtraining. A positive *R*^2^ value and negative *Q*^2^ value indicate a reliable, non-overtrained model. Variable importance in projection (VIP) was employed to identify key markers contributing significantly to discrimination (23). IBM SPSS software (Version 26.0, Armonk, NY, USA) was utilized to conduct significance analysis of differences, whereas graphical representations were executed using the Apps plugin in Origin software (Version, 2021 Pro; OriginLab Corp., Northampton, MA, USA).

## 3 Results and discussion

### 3.1 Texture properties

The TPA is a method to mimic the mechanical actions of the mouth and measure food texture characteristics without bias (24). Therefore, to study the influence of different drying processes on texture characteristics of black sweet corn, a comprehensive TPA, including hardness, frangibility, chewiness, and thickness, was conducted (Table 1).

**Table 1:** Effect of drying methods on the texture of black sweet corn kernels.

Drying methods	Thickness (mm)	Hardness (gf)	Frangibility (gf)	Chewiness (gf·s)
Fresh	4.173 ± 0.223^b^	202.491 ± 40.438^c^	–	149.352 ± 22.318^d^
FD	5.021 ± 0.671^a^	591.935 ± 154.755^b^	290.466 ± 90.450^b^	367.871 ± 82.661^c^
VD	5.280 ± 0.380^a^	67.816 ± 21.984^c^	11.003 ± 4.127^c^	72.267 ± 29.092^d^
HD	2.026 ± 0.195^c^	2,098.879 ± 186.711^a^	690.558 ± 93.339^a^	1,096.552 ± 186.781^a^
MD	5.137 ± 0.961^a^	679.555 ± 173.414^b^	55.813 ± 11.946^c^	577.234 ± 98.108^b^

Different superscript letters in the same column indicate significant differences (*p* < 0.05).

The thickness measured at horizontal corn grain placement can measure the deformation degree of corn after drying. This thickness was significantly greater in the FD, VD, and MD samples than in the fresh samples, with no significant difference (*p* < 0.05) among the former three groups. However, the HD samples showed obvious shrinkage. In FD and VD, the high vacuum conditions promote crystalline glass transition, which supports the structure and prevents collapse. In MD, rapid drying and surface hardening by microwave radiation similarly enhanced structural rigidity and restrained shrinkage (4). Hardness and chewiness exhibited similar patterns, from (Table 1). Chewiness reflects the energy required to transform food from a chewable state to a swallowing state, and it integrates the sample’s continuous resistance to chewing. Its value is the product of hardness, cohesion, and elasticity. Thus, it is positively correlated with a change in hardness (25). The hardness and chewiness values of the VD samples were the smallest among all the samples, with no significant differences (*p* < 0.05) with the fresh samples. The lower the hardness, the less effort it takes to chew. The frangibility of the VD and MD samples was significantly lower than that of the FD and HD samples (*p* < 0.05). Among all samples, the HD sample exhibited the highest frangibility, coupled with a relatively hard texture. These results suggest that the HD process is less suitable for black sweet corn (20).

### 3.2 Color

**Figure 1:**
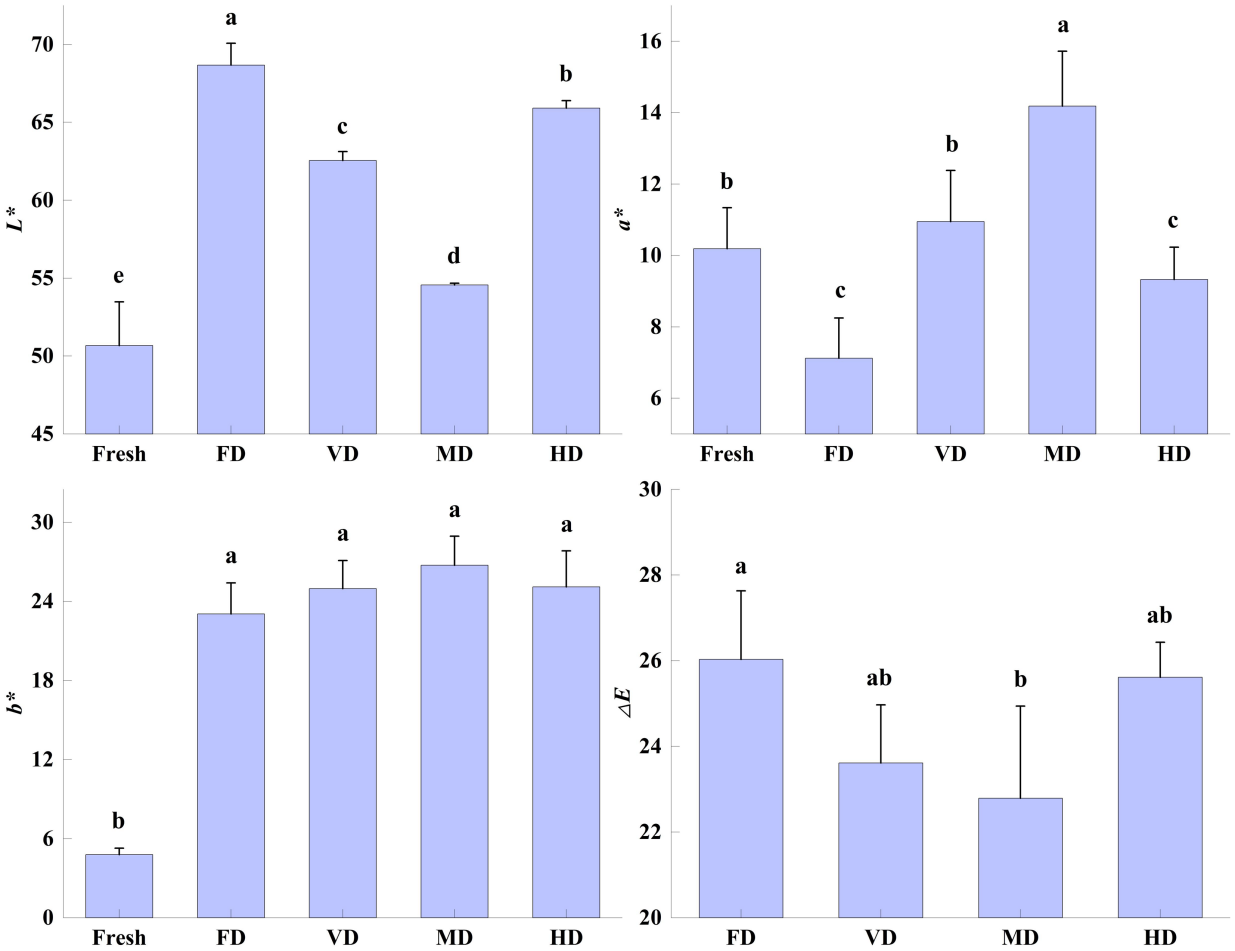
Effect of drying methods on the color of black sweet corn. Different letters represent significant differences (*p* < 0.05) among groups.

Color is an important attribute in evaluating the merit of food products, affecting the consumer’s choice and produce value. Different drying methods can change the color of dried products (20). The effect of drying methods on the color parameters of black sweet corn (L*, a*, b*, and Δ*E*) was highly significant (*p* < 0.05) (Figure 1).

After drying, the L* and b* values of the corn samples increased significantly (*p* < 0.05), while changes in a* values were inconsistent. This may be the result of variations in the oxidation and degradation of flavonoids (12). The L* value of the FD samples was the highest (68.67 ± 1.41), followed by the HD samples (65.92 ± 0.47), VD samples (62.55 ± 0.57), and MD samples (54.56 ± 0.12). The b* values of the corn samples after the four drying treatments were not significantly different from each other. Among all the samples, the b* value of the MD samples was the highest and that of the FD samples was the smallest. This could be the result of the differences in the heat treatment time, temperature, and degree of contact with gasses during the drying process, resulting in various pigment changes (11, 12). Similarly, the highest L* value and the smallest b* value also appeared in FD-dried red radish (14).

The a* value of the VD samples was the closest to that of the fresh samples, with no significant difference. This may be due to the vacuum inhibiting enzymatic browning and occurrence of the Maillard reaction. However, the a* value of the FD samples, which too were under low pressure, was significantly lower than the fresh samples. This might be the result of long drying times (27 h) and phased temperature changes (26). Compared with the fresh samples, the a* values of the FD and HD samples were significantly low, whereas those of the MD samples were significantly high. The a* values of the VD samples were not significantly different from those of the fresh corn samples.

Regarding the color difference (Δ*E*), only the Δ*E* values of the FD and MD samples showed significant differences (*p* < 0.05), with Δ*E* values of the FD samples being the highest among all the samples. Similar findings have been reported during the FD of yellow sweet corn (20) and sea buckthorn pomace (12).

The MD samples exhibited superior resistance to color change compared with the other drying methods (19). The black sweet corn color is mainly imparted by anthocyanins and internal enzymes. The drying temperature during MD was higher than the other treatments; however, the drying time was short, with less exposure to air. This resulted in decreased enzyme activity and redox homoeostasis in the corn samples. Therefore, the anthocyanin content was relatively higher than in the other treatments, with a high acylation degree, leading to small black sweet corn color changes (10).

### 3.3 RR

The RR is an important indicator in evaluating the dried product and can characterize the degree of destruction in the material structure due to drying. A high RR corresponds to better product quality, indicating minimal damage to the product structure (27).

**Figure 2:**
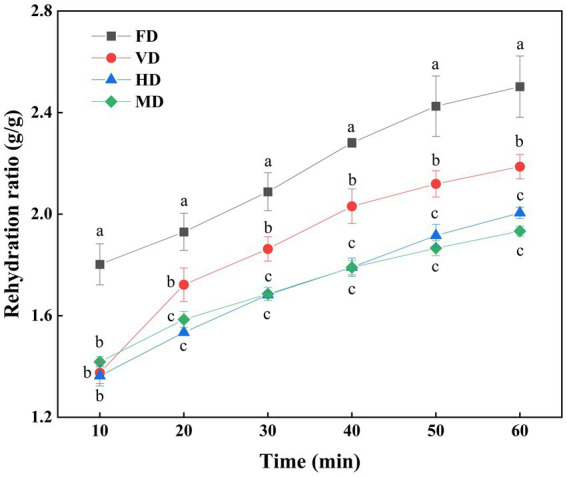
Rehydration ratio (RR) of black sweet corn processed by different drying methods. Data are presented as mean ± SD (*n* = 3). Different letters at the same time reveal significant differences (*p* < 0.05).

Figure 2 compares the complex water ratios of black sweet corn resulting from different drying methods. The pore size distribution in the internal structure of the dried products significantly influences both apparent density and rehydration properties (28). The effect of different drying methods on the RR of dried black sweet corns was closely related to microstructural changes, thus determining the macroscopic properties (26).

Overall, the FD samples exhibited the highest water absorption capacity followed by the VD samples, whereas the HD and MD samples exhibited the lower values among all the samples at the same immersion duration. This difference can be attributed to the formation of a honeycomb tissue structure in the FD and VD samples dried under vacuum. Conversely, the MD and HD samples formed a dense structure, resulting in lower water absorption capacity than the other samples (4). The water rehydration capacity is significantly influenced by the bulk density, porosity, and specific volume of the product, and increased porosity during the drying process enhances water rehydration performance (29).

### 3.4 Anthocyanins

Anthocyanins are important secondary metabolites in many fruits, and they are natural pigments with significant biological functions. However, anthocyanins are susceptible to environmental and processing factors, resulting in poor storage and processing stability (30).

**Figure 3:**
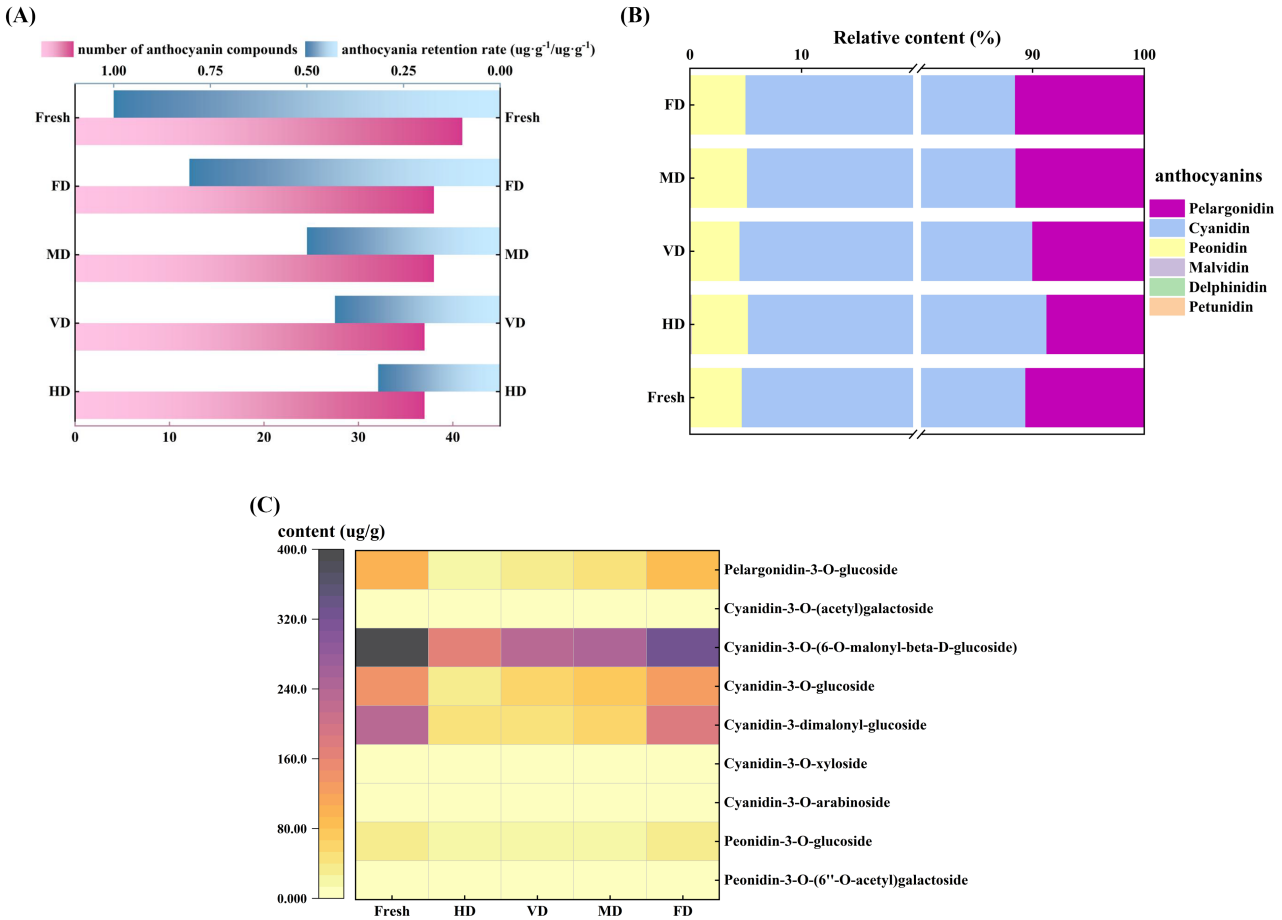
Effect of different drying methods on anthocyanins of black sweet corn. **(A)** Numbers of different types of anthocyanins detected and anthocyanin retention rate under different drying treatments. **(B)** Relative content (%) of the six anthocyanin components in different samples. **(C)** Heatmap of major anthocyanin compounds in different black sweet corn samples.

The fresh samples contained 43 anthocyanin components, and the anthocyanin diversity was reduced to 38, 38, 37, and 37, respectively, by FD, MD, VD, and HD process (Figure 3A). The anthocyanin components, namely, delphinidin-3-O-glucoside, malvidin-3-O-(6-O-acetyl) glucoside, cyanidin-3-O-rhamnoside, pelargonidin-3-O-sophoroside-5-O-glucoside, and pelargonidin-3-O-rutinoside-5-O-glucoside, and others, which were low in the fresh sample, were lost during the drying process and were not detected in the dried product.

Six anthocyanins commonly found in foods—pelargonidin, cyanidin, delphinidin, peonidin, petunidin, and malvidin (31)—were detected in this study. Among these, cyanidin (83.35–86.03%), pelargonidin (8.72–11.54%), and peonidin (4.36–5.35%) were the predominant anthocyanins (Figure 3B), which aligned with the findings reported by Chuntakaruk et al. (32). Furthermore, the primary anthocyanin components consisted of pelargonidin-3-O-glucoside, cyanidin-3-O-(6-O-malonyl-β-D-glucoside), cyanidin-3-O-glucoside, cyanidin-3-dimalonyl-glucoside, peonidin-3-O-glucoside, and others (Figure 3C). This study findings corroborate with the anthocyanin identification findings in purple corn by Ren and Giusti (31) and Ziyan tea wine by Lin et al. (15). However, cyanidin 3-O-glucoside—abundant in mulberries (13)—was detected at considerably low levels in black sweet corn samples, owing to the differences in anthocyanin composition (33). Zhou et al. (13) demonstrated that under identical temperatures (60 and 75 °C), the retention rates of cyanidin-3-O-glucoside (C3G) and cyanidin-3-O-rutinoside (C3R) in mulberry fruit under VD were consistently higher than after HD. This indicates that a low-oxygen atmosphere may help alleviate anthocyanin degradation during thermal processing. Therefore, it is recommended that minimizing oxygen exposure during drying promotes anthocyanin retention.

It has been reported that various drying treatments can reduce the anthocyanin content in purple corn (8). Among these, HD resulted in the least anthocyanin loss (5.9%). The three drying methods of sun drying, MD, and FD exhibited comparable anthocyanin losses in a range of 29.4–30.4%. Differences between these reports and the present study findings may be attributed to the relatively crude anthocyanin detection method (differential method) used in the reported studies (9).

Laokuldilok and Kanha (7) investigated that FD anthocyanin powders of black glutinous rice bran showed better anthocyanin retention, bulk density, angle of repose, and process yield than the anthocyanin powders of spray dried (SD) samples. The anthocyanin retention rate in the FD and SD samples was 72 and 48–72%, respectively.

The FD, MD, and VD samples demonstrated high anthocyanin retention rates (Figure 3A), which was primarily attributed to their superior drying efficiency, reduced heat exposure, or short contact time between black sweet corn and oxygen. These factors collectively minimized the oxidation of anthocyanin compounds, leading to elevated retention levels (13). Comparable findings have been reported on mulberry (*Morus alba* L.) fruit (13) and purple potato (10) drying, where FD has been identified as the optimal method for preserving anthocyanins in these produce. Temperature significantly affects anthocyanin stability. Thus, extended heating duration and elevated temperatures can intensify the thermal degradation of anthocyanins (8). Furthermore, the unsaturated components of anthocyanins render them highly susceptible to oxygen. Oxygen accelerates anthocyanin degradation by impairing the function of oxidative enzymes, such as polyphenol oxidase (34).

The low-temperature and vacuum conditions of FD created a favorable environment for anthocyanin stability, thus FD was widely regarded as a valid method for protecting anthocyanins during dehydration (14). At lower power with short-term MD treatment, the main effect on anthocyanins was the hydrolysis of anthocyanin glycosides to their aglycones, which resulted in a relatively small reduction in the total anthocyanin content (19).

### 3.5 Volatile compounds

Volatile compounds are essential determinants of overall aroma characteristics of food and often influence human preferences or aversions toward taste (22). To reflect the effects of different drying methods on the aroma characteristics of black sweet corn, the volatile components of corn samples were analyzed by HS-SPME-GC–MS (6) (Table 2). Sixty seven aroma components were detected, encompassing 10 alcohols, 1 aldehyde, 4 pyrazines, 7 aldehydes, 5 acids, 5 ketones, 20 esters, 12 alkanes and olefins, and 3 other compounds. The volatile compound content after drying treatments was higher than that in the fresh samples (Table 2). Notably, the FD sample exhibited a significant increase (*p* < 0.05), with a relative volatile compound content reaching 158.65 ± 39.21 μg/g, whereas no significant increases were observed in the other drying treatments, with relative volatile compound contents of 97.81 ± 22.15 μg/g (HD), 79.99 ± 9.76 μg/g (VD), and 67.50 ± 9.62 μg/g (MD).

**Table 2:** Determination of volatile components and their relative contents in black sweet corn samples by HS-SPME-GC-MS.

Class (No.)	CAS	Compound	Retention time (min)	Relative contents (μg/g)				
				Fresh	VD	FD	MD	HD
Alcohols								
1	24347-58-8	(Z)-2,3-butanediol	27.78	1.16 ± 0.13^c^	7.94 ± 0.47^b^	11.13 ± 1.85^a^	7.54 ± 2.61^b^	8.83 ± 0.36^ab^
2	111-70-6	1-Heptanol	24.39	0.58 ± 0.08	ND	ND	ND	ND
3	3391-86-4	1-Octen-3-ol	24.10	1.67 ± 0.20^ab^	1.21 ± 0.19^c^	1.92 ± 0.27^a^	1.55 ± 0.12^bc^	1.41 ± 0.01^bc^
4	104-76-7	2-Ethylhexanol	25.76	0.31 ± 0.05^b^	0.17 ± 0.05^c^	0.47 ± 0.02^a^	0.22 ± 0.02^c^	0.39 ± 0.08^ab^
5	470-82-6	Eucalyptol	15.89	0.44 ± 0.04^b^	ND	1.57 ± 0.12^a^	ND	ND
6	60-12-8	Phenethyl alcohol	44.26	0.52 ± 0.05^ab^	0.27 ± 0.05^b^	0.44 ± 0.01^ab^	0.24 ± 0.00^b^	0.97 ± 0.68^a^
7	18409-17-1	(E)-2-octen-1-ol	30.88	0.40 ± 0.08	ND	ND	ND	ND
8	78-70-6	Linalool	18.09	0.19 ± 0.05^a^	0.28 ± 0.09^a^	0.32 ± 0.14^a^	0.39 ± 0.13^a^	ND
9	64-17-5	Ethyl alcohol	8.31	15.53 ± 1.50^c^	2.44 ± 0.68^d^	30.55 ± 4.27^a^	23.75 ± 1.64^b^	1.55 ± 0.03^d^
10	111-27-3	1-Hexanol	20.42	3.32 ± 0.46^a^	0.22 ± 0.04^c^	0.45 ± 0.03^c^	0.99 ± 0.01^b^	0.21 ± 0.01^c^
Subtotal				24.10 ± 2.28^c^	12.53 ± 0.35^d^	46.84 ± 5.04^a^	34.66 ± 4.44^b^	13.34 ± 0.37^d^
Phenols								
1	7786-61-0	4-Vinylguaiacol	54.23	0.12 ± 0.03^c^	0.34 ± 0.11^b^	ND	0.30 ± 0.04^b^	0.64 ± 0.15^a^
Pyrazines								
1	14667-55-1	2,3,5-Trimethylpyrazine	22.80	ND	ND	ND	ND	1.22 ± 0.07
2	108-50-9	2,6-Dimethylpyrazine	19.86	ND	0.26 ± 0.01^b^	ND	ND	1.14 ± 0.18^a^
3	13067-27-1	2,6-Diethylpyrazine	25.07	ND	ND	0.21 ± 0.03^b^	ND	0.43 ± 0.02^a^
4	13925-09-2	2-Methyl-6-vinylpyrazine	25.98	ND	ND	ND	0.18 ± 0.01	ND
Subtotal					0.26 ± 0.01^b^	0.21 ± 0.03^b^	0.18 ± 0.01^b^	2.79 ± 0.27^a^
Aldehydes								
1	5445-77-2	2-Methyl-3-phenylpropanal	38.58	ND	ND	ND	1.06 ± 0.11	ND
2	498-60-2	3-Furaldehyde	24.56	ND	0.11 ± 0.02^b^	ND	ND	0.36 ± 0.04^a^
3	100-52-7	Benzaldehyde	27.25	0.13 ± 0.01^d^	0.33 ± 0.03^b^	0.32 ± 0.05^bc^	0.26 ± 0.02^c^	0.62 ± 0.05^a^
4	18829-56-6	(E)-2-Nonenal	27.93	1.00 ± 0.18^a^	0.48 ± 0.16^b^	0.50 ± 0.14^b^	0.43 ± 0.09^b^	0.43 ± 0.04^b^
5	2548-87-0	(E)-2-Octenal	23.62	0.45 ± 0.08^a^	ND	ND	ND	0.17 ± 0.00^b^
6	18829-55-5	(E)-2-Heptenal	19.60	0.25 ± 0.05	ND	ND	ND	ND
7	124-19-6	Nonanal	22.13	0.24 ± 0.07^d^	0.74 ± 0.08^ab^	0.40 ± 0.03^c^	0.85 ± 0.05^a^	0.68 ± 0.07^b^
Subtotal				2.09 ± 0.36^bc^	1.65 ± 0.29^cd^	1.22 ± 0.06^d^	2.59 ± 0.28^a^	2.26 ± 0.18^ab^
Acids								
1	591-81-1	4-Hydroxybutanoic acid	31.71	ND	0.27 ± 0.01^b^	ND	ND	0.52 ± 0.01^a^
2	111-14-8	Heptanoic acid	46.49	0.69 ± 0.18	ND	ND	ND	ND
3	112-05-0	Nonanoic acid	53.89	0.36 ± 0.07^a^	0.17 ± 0.01^b^	ND	ND	ND
4	64-19-7	Acetic acid	23.89	7.47 ± 2.68^a^	4.45 ± 0.56^b^	4.28 ± 0.49^b^	1.18 ± 0.08^c^	8.74 ± 0.85^a^
5	142-62-1	Hexanoic acid	41.25	6.88 ± 1.06^a^	2.75 ± 0.05^c^	1.88 ± 0.15^c^	1.39 ± 0.02^c^	4.90 ± 2.09^b^
Subtotal				15.40 ± 3.77^a^	7.64 ± 0.51^b^	6.15 ± 0.63^bc^	2.57 ± 0.10^c^	14.16 ± 2.40^a^
Ketones								
1	28564-83-2	2,3-Dihydro-3,5-dihydroxy-6-methyl-4h-pyran-4-one	55.72	ND	0.45 ± 0.25^a^	ND	ND	0.64 ± 0.07^a^
2	38284-27-4	3,5-Octadien-2-one	27.14	0.08 ± 0.01^c^	0.21 ± 0.01^b^	0.48 ± 0.07^a^	ND	ND
3	513-86-0	3-Hydroxy-2-butanone	18.14	0.37 ± 0.01^d^	1.59 ± 0.12^c^	2.58 ± 0.25^a^	1.50 ± 0.31^c^	2.04 ± 0.09^b^
4	1669-44-9	3-Octen-2-one	22.75	0.09 ± 0.00^b^	ND	0.63 ± 0.09^a^	ND	ND
5	19322-27-1	4-Hydroxy-5-methyl-3-furanone	52.41	ND	0.74 ± 0.20^b^	0.33 ± 0.01^c^	ND	1.78 ± 0.09^a^
Subtotal				0.54 ± 0.01^d^	2.99 ± 0.54^b^	4.01 ± 0.37^a^	1.50 ± 0.31^c^	4.46 ± 0.21^a^
Hydro-carbons								
1	20959-33-5	7-Methylheptadecane	19.54	ND	0.28 ± 0.01^b^	0.40 ± 0.05^a^	0.42 ± 0.01^a^	ND
2	18869-72-2	9-Methylheptadecane	14.24	ND	2.41 ± 0.18^ab^	2.40 ± 0.26^ab^	1.47 ± 1.22^b^	3.03 ± 0.18^a^
3	112-40-3	Dodecane	15.31	ND	ND	ND	5.02 ± 0.46	ND
4	544-76-3	Hexadecane	22.31	0.28 ± 0.01^b^	0.76 ± 0.09^a^	0.65 ± 0.34^a^	ND	0.98 ± 0.10^a^
5	629-78-7	Heptadecane	22.31	0.10 ± 0.00^b^	1.07 ± 0.03^a^	1.05 ± 0.02^a^	0.96 ± 0.11^a^	ND
6	629-59-4	Tetradecane	22.32	0.12 ± 0.01^c^	3.27 ± 0.38^b^	4.12 ± 0.48^a^	ND	3.84 ± 0.12^ab^
7	71138-64-2	3-Methylene-undecane	16.44	ND	0.83 ± 0.25^ab^	0.97 ± 0.01^a^	0.66 ± 0.07^b^	1.01 ± 0.15^a^
8	593-45-3	Octadecane	21.08	ND	ND	1.09 ± 0.15^a^	0.96 ± 0.02^a^	0.99 ± 0.04^a^
9	629-20-9	1,3,5,7-Cyclooctatetraene	17.09	0.33 ± 0.01^d^	0.81 ± 0.00^b^	0.80 ± 0.01^b^	0.95 ± 0.07^a^	0.59 ± 0.02^c^
10	61142-36-7	3-Ethyl-2-methyl-1,3-hexadiene	23.20	2.90 ± 0.33	ND	ND	ND	ND
11	100-42-5	Styrene	17.09	0.38 ± 0.02^b^	0.65 ± 0.02^a^	0.66 ± 0.05^a^	ND	0.61 ± 0.01^a^
12	4180-23-8	Anethole	40.47	ND	ND	1.12 ± 0.20	ND	ND
Subtotal				4.10 ± 0.37^c^	10.08 ± 0.87^b^	13.28 ± 0.86^a^	10.54 ± 1.42^b^	11.06 ± 0.34^b^
Esters								
1	6846-50-0	2,2,4-Trimethyl-1,3-pentanediol diisobutyrate	43.17	0.34 ± 0.07^b^	0.47 ± 0.35^b^	0.80 ± 0.45^b^	ND	1.53 ± 0.01^a^
2	67160-14-9	Methyl N-hydroxybenzenecarboximidate	36.96	0.43 ± 0.07^a^	0.13 ± 0.00^c^	0.16 ± 0.01^c^	0.27 ± 0.04^b^	ND
3	101-97-3	Ethyl phenylacetate	38.55	0.23 ± 0.01^a^	0.23 ± 0.05^a^	ND	ND	0.20 ± 0.02^a^
4	1937-62-8	Methyl trans-9-octadecenoate	46.21	1.96 ± 0.04^b^	8.75 ± 0.01^a^	10.45 ± 4.88^a^	0.32 ± 0.00^b^	10.18 ± 3.10^a^
5	10030-74-7	Methyl palmitelaidate	55.41	ND	0.79 ± 0.01^a^	1.66 ± 0.79^a^	ND	0.71 ± 0.31^a^
6	6114-18-7	(E)-9-Octadecenoic acid ethyl ester	50.67	1.37 ± 1.28^a^	0.71 ± 0.60^a^	0.35 ± 0.01^a^	0.70 ± 0.10^a^	0.95 ± 0.01^a^
7	408-14-0	Fluoroacetic acid dodecyl ester	16.71	ND	0.61 ± 0.08^b^	0.54 ± 0.07^b^	ND	0.76 ± 0.05^a^
8	106-30-9	Ethyl heptanoate	19.82	0.20 ± 0.11^b^	ND	0.60 ± 0.01^a^	ND	ND
9	106-70-7	Methyl caproate	14.83	ND	ND	1.19 ± 0.09^a^	ND	0.72 ± 0.03^b^
10	1731-84-6	Methyl nonanoate	26.02	ND	0.60 ± 0.07^c^	1.03 ± 0.18^b^	ND	1.43 ± 0.12^a^
11	124-10-7	Methyl myristate	49.15	ND	0.16 ± 0.01^b^	0.52 ± 0.12^a^	ND	0.20 ± 0.01^b^
12	7132-64-1	Methyl pentadecanoate	52.57	ND	0.06 ± 0.00^c^	0.18 ± 0.01^b^	ND	0.31 ± 0.02^a^
13	111-11-5	Octanoic acid methyl ester	21.91	0.10 ± 0.01^c^	1.57 ± 0.10^b^	2.84 ± 0.32^a^	0.24 ± 0.01^c^	2.91 ± 0.06^a^
14	106-32-1	Ethyl caprylate	23.69	0.44 ± 0.41^b^	0.88 ± 0.22^ab^	1.33 ± 0.14^a^	1.22 ± 0.07^a^	0.95 ± 0.66^ab^
15	112-63-0	Methyl linoleate	51.25	3.01 ± 0.6^a^	7.84 ± 2.20^a^	7.98 ± 1.71^a^	0.70 ± 0.54^b^	5.63 ± 2.06^a^
16	544-35-4	Ethyl linoleate	53.06	1.31 ± 0.89^b^	2.84 ± 0.02^b^	9.49 ± 5.88^a^	2.69 ± 1.94^b^	5.07 ± 1.28^ab^
17	111-62-6	Ethyl oleate	50.56	0.56 ± 0.25^a^	0.61 ± 0.00^a^	0.99 ± 0.73^a^	0.65 ± 0.33^a^	ND
18	123-66-0	Ethyl hexanoate	16.32	1.37 ± 0.65^b^	1.05 ± 0.81^b^	1.60 ± 0.30^b^	0.61 ± 0.01^b^	2.75 ± 0.75^a^
19	112-39-0	Methyl hexadecanoate	54.91	3.37 ± 0.36^bc^	12.77 ± 4.42^bc^	31.51 ± 12.57^a^	2.38 ± 0.45^c^	14.62 ± 3.72^b^
20	628-97-7	Ethyl palmitate	55.63	4.87 ± 0.32^b^	4.09 ± 0.30^b^	11.22 ± 5.62^a^	4.89 ± 0.89^b^	2.87 ± 1.27^b^
Subtotal				19.58 ± 3.66^b^	44.18 ± 8.84^b^	84.45 ± 33.23^a^	14.68 ± 3.97^b^	48.40 ± 18.98^b^
Others								
1	67-68-5	Sclerosol	30.03	ND	0.46 ± 0.02^b^	ND	ND	0.80 ± 0.06^a^
2	140-67-0	Estragole	33.39	0.20 ± 0.02^b^	ND	2.34 ± 0.51^a^	0.28 ± 0.03^b^	ND
3	3777-69-3	2-Pentylfuran	16.17	0.32 ± 0.05^b^	0.20 ± 0.01^c^	0.15 ± 0.00^c^	0.50 ± 0.04^a^	0.52 ± 0.02^a^
Subtotal				0.52 ± 0.06^c^	0.66 ± 0.03^c^	2.48 ± 0.51^a^	0.77 ± 0.08^c^	1.32 ± 0.06^b^
Total				66.32 ± 3.02^b^	79.99 ± 9.76^b^	158.65 ± 39.21^a^	67.50 ± 9.62^b^	97.81 ± 22.15^b^

ND, not detected. Different superscript letters in the same column represent significant differences among groups (*p* < 0.05).

Among the aldehydes identified in the black sweet corn samples, (E)-2-heptenal, nonanal, (E)-2-octenal, 3-furaldehyde, Benzaldehyde, (E)-2-nonenal, and 2-methyl-3-phenylpropanal were detected. The relative proportions of those identified aldehydes ranged from 1.22 ± 0.06 to 2.59 ± 0.28 μg/g. Although the overall aldehyde content was relatively low, their low odor thresholds contributed to significant enhancement of overall aroma. Aldehydes, primarily originating from typical compounds formed via lipid oxidation, make significant contributions to sweet, floral, and fruity aromas (35). After MD treatment, the aldehyde content significantly increased, whereas the FD treatment significantly decreased it. This discrepancy may be attributed to temperature being an important factor influencing aldehyde formation. Elevated temperatures in MD promote the Maillard reaction and Strecker degradation, leading to increased generation of aldehydes. Furthermore, an aerobic environment facilitates the oxidation of unsaturated fatty acids, thereby yielding aldehydes derived from oxidative processes, such as nonanal (36, 37). Ketones are typical compounds primarily produced through lipid oxidation, including 3-hydroxy-2-butanone, 3-octen-2-one, 3,5-octadien-2-one, 4-hydroxy-5-methyl-3-furanone, and 2,3-dihydro-3,5-dihydroxy-6-methyl-4h-pyran-4-one. These ketones possess diverse aroma characteristics, including green, floral, fruity, and butter scents (23). All the four dehydration processes, particularly FD and HD treatments, significantly increased the ketone content. Alcohols are generally formed by the reduction of aldehydes or ketones, and their aroma correlates with the number of carbon atoms in the compound. Alcohols identified using GC–MS, include ethyl alcohol, eucalyptol, 1-hexanol, 1-octen-3-ol, 1-heptanol, 2-ethylhexanol, (Z)-2,3-butanediol, linalool, (E)-2-octen-1-ol, and phenethyl alcohol. Among these, ethyl alcohol and (Z)-2,3-butanediol exhibited relatively high contents; however, their higher odor thresholds resulted in lesser contributions to the overall flavor (23). Esters are primarily formed through esterification reactions between alcohols and acidic substances (4). Among the volatile compounds detected in the corn samples, esters were the most diverse, mainly comprising methyl caproate, ethyl hexanoate, ethyl heptanoate, octanoic acid methyl ester, ethyl caprylate, methyl nonanoate, methyl trans-9-octadecenoate, methyl myristate, ethyl oleate, methyl linoleate, ethyl linoleate, methyl hexadecanoate, methyl palmitelaidate, and ethyl palmitate. These esters exhibited rich aromas, predominantly characterized by fruity, grassy, floral, waxy, fatty, and creamy notes. Except the MD treatment, the VD, FD, and HD treatments increased the ester content, with FD treatment notably enhancing and enriching the floral and fruity aromas of the product (Table 2).

The types of ester compounds detected were few, with thermally sensitive compounds such as methyl linoleate being significantly lower (*p* < 0.05), resulting in the lowest ester content in the MD sample. This can be attributed to the oxidation and subsequent decomposition of esters during repeated heating, leading to the formation of volatile carbonyl compounds such as aldehydes (Table 2) (37). Pyrazines were not detected in the fresh corn; however, these were detected in the four drying samples. Among all the samples, the highest pyrazine content was observed in the HD samples (2.79 ± 0.27 μg/g), followed by the VD samples (0.26 ± 0.01 μg/g). Pyrazine compounds exhibited typical roasted and nutty aromas (38). This observation suggests that prolonged heat treatment promotes pyrazine compound formation. As shown in Table 2, drying treatments produced hydrocarbons such as 9-methylheptadecane, dodecane, and octadecane. Similar phenomena were also observed in studies by Zhang et al. (22) and Yao et al. (4). All four drying methods significantly increased the relative content of hydrocarbons (*p* < 0.05). Lipid oxidation degradation and Maillard reactions during drying can generate alkanes and alkenes (22, 23). Most of these hydrocarbons possess high odor thresholds and contribute little to direct flavor perception.

**Figure 4:**
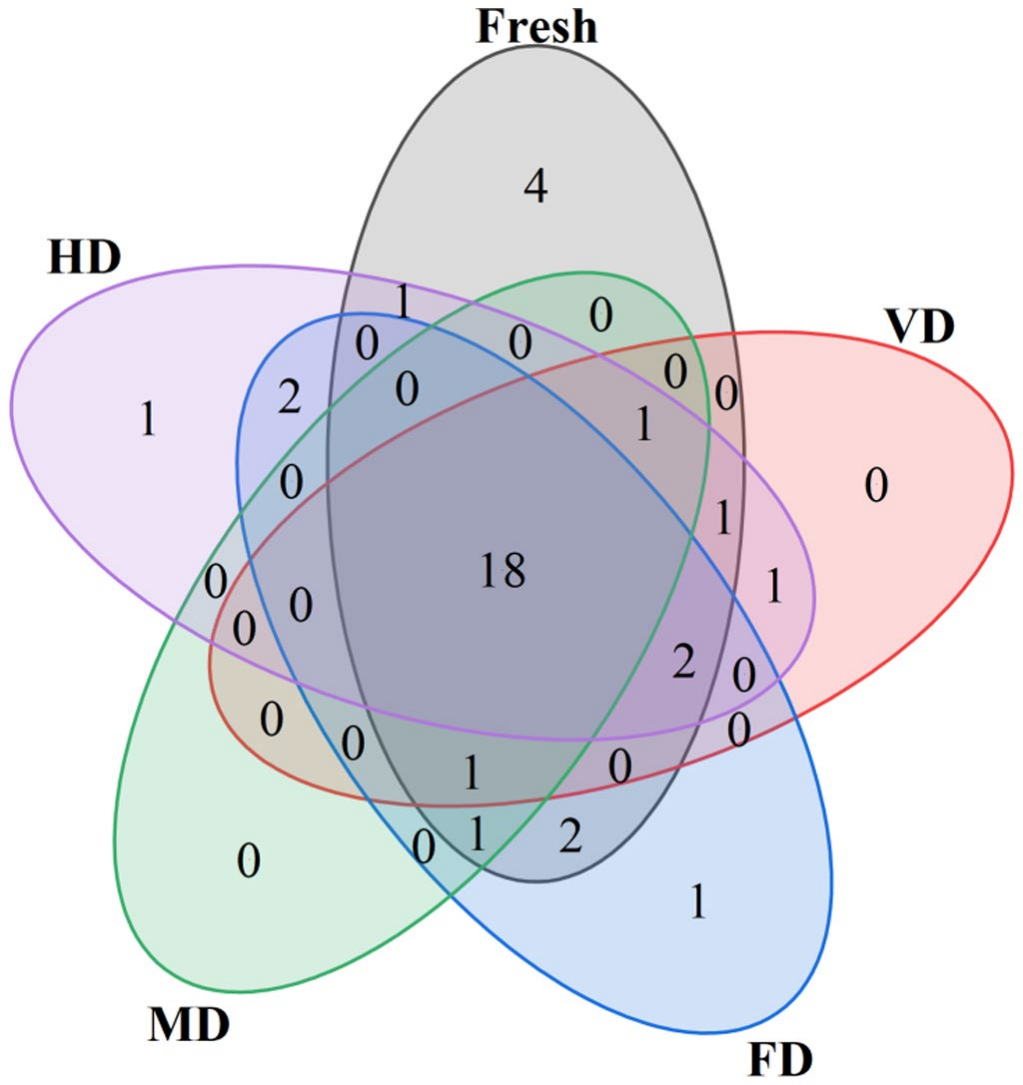
Venn plot of volatile compounds with odor activity value (OAV) greater than 1 in corn samples.

The food flavor is primarily contributed by key volatile compounds. Odor activity value (OAV) represents the contribution of individual aroma compounds to the overall aroma. It is generally considered that compounds with an OAV > 1 have a significant impact on the overall flavor (39) and are regarded as key flavor compounds. The number of calculable OAV > 1 compounds in the fresh, VD, FD, WB, and HD samples were 31, 24, 27, 21, and 27, respectively (Figure 4).

**Figure 5:**
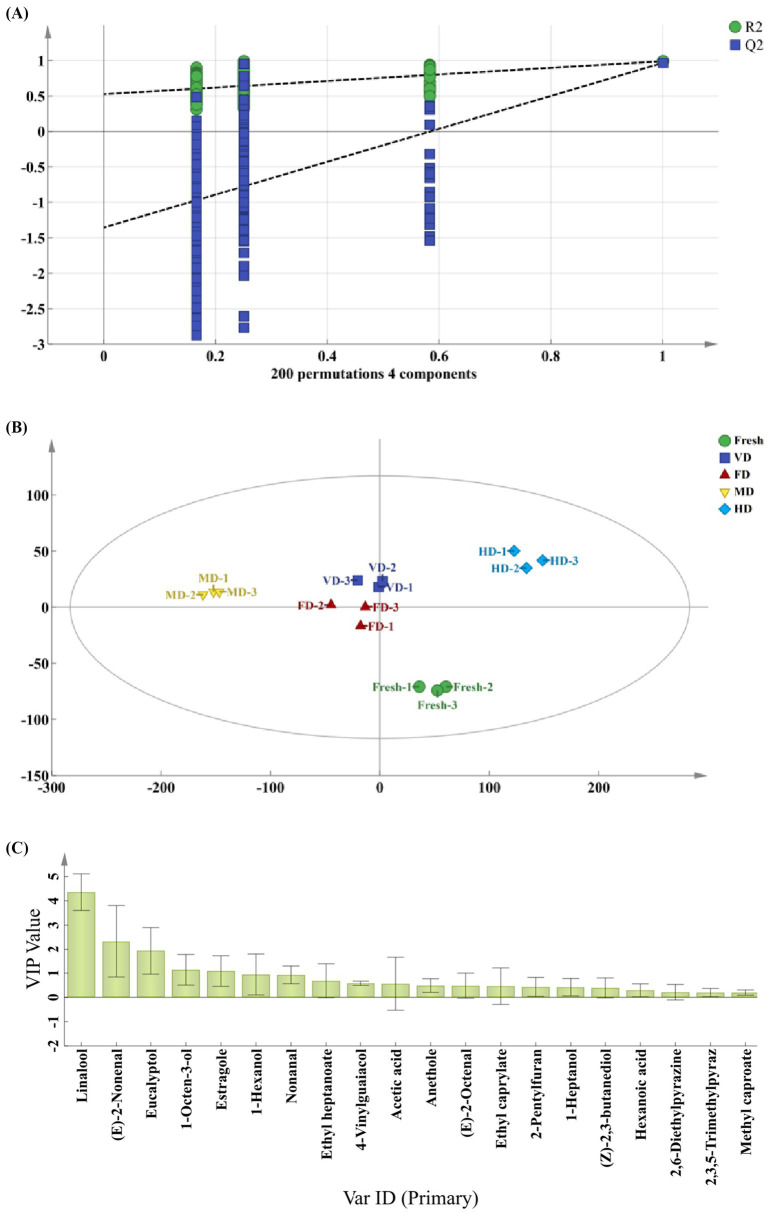
Orthogonal partial least squares-discriminant analysis (OPLS-DA) of aroma substances in five corn samples. **(A)** Principal component analysis (PCA) score plot. **(B)** Permutation test at 200 times. **(C)** Variable importance in projection (VIP) plot.

To screen the major flavor compounds with discriminatory contributions to corn aroma profiles, OPLS-DA was conducted on key flavor compounds with OAV > 1 using the previously reported method (6), and VIP values were calculated (Figure 5). The *R*^2^Y value of the samples was 0.927, closely approaching 1 and fell within the 95% confidence interval, indicating effective differentiation between samples of different groups (Figure 5A). The reliability of the model was further validated via permutation tests. After 200 cross-validations, the *Q*^2^ regression line of the model intersected the *X*-axis, with all *Q*^2^ points positioned above the corresponding left-side points, and the intercept of the line crossing the *Y*-axis was under zero (Figure 5B). This suggested no overfitting, confirming the validity of the established OPLS-DA model, which effectively reflected the differences in volatile compounds among the samples (39). The VIP values in the OPLS-DA model quantify the influence intensity and explanatory capacity of component accumulation differences on sample classification. A high VIP value indicates a great difference in volatile flavor compounds among corn samples and a more pronounced contribution to sample differentiation. A VIP > 1 is generally considered indicative of an important variable, representing a differential flavor compound (6, 23). The key markers with VIP > 1 included linalool, (E)-2-nonenal, eucalyptol, 1-octen-3-ol, estragole (Figure 5C).

(E)-2-nonenal and 1-octen-3-ol were identified as key aroma-active compounds in sweet corn subjected to thermal treatments (steaming, blanching, and roasting) at temperatures above 100 °C, both exhibiting low odor thresholds. (E)-2-nonenal was characterized by a green and tallow-like aroma, whereas 1-octen-3-ol contributed a mushroom-like odor to the thermal-processed corn (22). Compared with that in the fresh samples, the (E)-2-nonenal content decreased significantly in all the four drying methods. In dry-processed corn, the decrease in the (E)-2-nonenal content could be the result of lipoxygenase destruction, which promotes the oxidative cleavage of lipids to produce (E)-2-nonenal. As the degree of treatment increased, (E)-2-nonenal decreased more obviously until disappeared (22). Compared with that in the fresh samples, the relative 1-octen-3-ol content increased in the FD samples, whereas it decreased in all other samples, particularly in the VD samples (*p* < 0.05). This indicated that 1-octen-3-ol (with a low boiling point) evaporated more severely or lost with water vapors during the VD treatment. Linalool was identified as one of the major aroma-active compounds in steamed sweet potato (24), exhibiting citrus-like and camphor-like aromatic characteristics (6). In addition, estragole has been reported as a discriminatory aroma compound in Egyptian fennel, characterized by anise-like, fruity, and pungent notes (40), whereas eucalyptol contributes to camphor, cool, eucalyptus, and mint flavors. Overall, among the four dehydrated samples, the FD samples exhibited the highest linalool, (E)-2-nonenal, eucalyptol, 1-octen-3-ol, and estragole contents, followed by the MD samples.

## 4 Conclusion

Based on the above-mentioned results, it is apparent that HD, VD, FD, and MD had considerable effects on the color, RR, texture, anthocyanin and volatile compounds of black sweet corn. TPA revealed that FD, VD, and MD well maintained the morphology of the corn kernels, with VD additionally providing easier chewability. In contrast, HD significantly deteriorated the overall texture quality. The L* and b* values of the fresh corn samples were significantly improved by the FD and MD process. Moreover, MD exhibited the least color deviation from the fresh sample, which can be attributed to suppressed enzymatic browning and limited anthocyanin degradation. For rehydration properties, the FD and VD samples, characterized by their porous structures, demonstrated the strongest rehydration capacity. FD and MD exhibited better performance in preserving the major anthocyanin components, including cyanidin-3-O-(6-O-malonyl-beta-D-glucoside), pelargonidin-3-O-glucoside, cyanidin-3-dimalonyl- glucoside and et al., along with the total anthocyanin content and retention rates, compared to other drying methods. Moreover, the dehydration process enriched the aroma attributes of the black sweet corn. Statistical analysis identified linalool, (E)-2-nonenal, eucalyptol, 1-octen-3-ol, and estragole as key discriminant aroma markers (VIP > 1) across all samples. Among the four dehydrated samples, FD exhibited the highest content of these five compounds, followed by MD. Overall, the FD and MD samples performed the best performance in terms of simulated ready-to-eat property, core nutritional indicators such as anthocyanins, and aroma. This suggests that FD and MD can be used as effective ways to dry black sweet corn. However, FD has limitations for industrial use because it requires expensive equipment and a long drying time. MD is a viable alternative as it consumes less energy, accelerates the drying process, and is suitable for large-scale production. However, further work is required to enhance our understanding of drying process regulation and its impact on product quality characteristics by diversifying material varieties of black sweet corn, integrating drying kinetics and consumer sensory evaluation. Such investigations will facilitate the optimization of drying technologies for black sweet corn, thereby promoting informed decision-making in production methodologies.

## Data Availability

The original contributions presented in the study are included in the article/supplementary material, further inquiries can be directed to the corresponding author.
